# Unified Methodology for the Primary Preclinical In Vivo Screening of New Anticoagulant Pharmaceutical Agents from Hematophagous Organisms

**DOI:** 10.3390/ijms25073986

**Published:** 2024-04-03

**Authors:** Maria A. Kostromina, Elena A. Tukhovskaya, Elvira R. Shaykhutdinova, Yuliya A. Palikova, Viktor A. Palikov, Gulsara A. Slashcheva, Alina M. Ismailova, Irina N. Kravchenko, Igor A. Dyachenko, Evgeniy A. Zayats, Yuliya A. Abramchik, Arkady N. Murashev, Roman S. Esipov

**Affiliations:** 1 Laboratory of Biopharmaceutical Technologies, Shemyakin and Ovchinnikov Institute of Bioorganic Chemistry, Russian Academy of Sciences, Miklukho-Maklaya Street, 16/10, 117997 Moscow, Russia; 2 Biological Testing Laboratory, Branch of Shemyakin and Ovchinnikov Institute of Bioorganic Chemistry, Russian Academy of Sciences, Pushchino, ProspektNauki, 6, 142290 Moscow, Russia

**Keywords:** activated partial thromboplastin time (APTT), prothrombin time (PT), fibrinogen, hemostasis, anticoagulant, variegin, heparin, dabigatran etexilate, mice, rat, bleeding, venous thrombosis

## Abstract

The development of novel anticoagulants requires a comprehensive investigational approach that is capable of characterizing different aspects of antithrombotic activity. The necessary experiments include both in vitro assays and studies on animal models. The required in vivo approaches include the assessment of pharmacokinetic and pharmacodynamic profiles and studies of hemorrhagic and antithrombotic effects. Comparison of anticoagulants with different mechanisms of action and administration types requires unification of the experiment scheme and its adaptation to existing laboratory conditions. The rodent thrombosis models in combination with the assessment of hemostasis parameters and hematological analysis are the classic methods for conducting preclinical studies. We report an approach for the comparative study of the activity of different anticoagulants in vivo, including the investigation of pharmacodynamics and the assessment of hemorrhagic effects (tail-cut bleeding model) and pathological thrombus formation (inferior vena cava stenosis model of venous thrombosis). The reproducibility and uniformity of our set of experiments were illustrated on unfractionated heparin and dabigatran etexilate (the most common pharmaceuticals in antithrombic therapy) as comparator drugs and an experimental drug variegin from the tick *Amblyomma variegatum*. Variegin is notorious since it is a potential analogue of bivalirudin (Angiomax, Novartis AG, Basel, Switzerland), which is now being actively introduced into antithrombotic therapy.

## 1. Introduction

Due to the widespread prevalence of thrombo-associated cardiovascular diseases, standard protocols for the use of antithrombotic drugs have been introduced in the therapeutic practice. Usually, antiplatelet agents, anticoagulants, and fibrinolytic drugs are used in therapy in various combinations. The biological activity of these pharmaceuticals is aimed at eliminating the corresponding endo- or exogenous pathological processes [1,2,3]. Moreover, due to the variability in blood flow conditions (blood pressure, blood flow speed) and characteristics of the endothelium, blood clots of different compositions form in the arteries and veins. Both types of blood clots (arterial and venous) consist of platelets and fibrin; however, clots formed in the arteries also include leukocytes (white blood clots), while those formed in the veins contain saturated red blood cells (red blood clots) [4]. Therefore, there are two types thrombosis depending on the localization of platelet-fibrin clot formation: arterial and venous thrombosis. The former is responsible for the development of ischemic stroke and acute myocardial infarction, while the latter causes deep vein thrombosis (DVT), pulmonary embolism (PE) and unstable angina [4,5]. At the same time, patients with venous thromboembolism are at high risk of developing arterial thrombosis and vice versa. One of the integral parts of thrombosis prevention and therapy is the administration of direct and indirect anticoagulants.

Indirect anticoagulants or vitamin K antagonists have many limitations despite being used in practice for more than 70 years due to their ease of administration [6]. Therefore, the majority of the current studies are dedicated to the search for new, effective direct anticoagulants, the action of which is aimed at direct, highly specific blocking of key factors of the blood coagulation system, mainly thrombin and factor Xa [7]. It is worth highlighting that the main indicator by which the pharmacological profile of new drugs is assessed is the likelihood of developing hemorrhagic complications. The most universal anticoagulant for the prevention and treatment of arterial and venous thromboembolism is unfractionated heparin (UFH) [8]. It is commonly prescribed to a broad variety of patients due to the multitude of its therapeutic effects: anticoagulant, antithrombotic, antiplatelet and antitumor activity. Other benefits of UFH include quick action after parenteral administration and simple monitoring methods. However, the administration of heparin is complicated by a variety of adverse side effects, including the likely occurrence of pathological bleeding and heparin-induced thrombocytopenia, which can result in development of severe forms of venous and/or arterial thrombosis of immune genesis [9]. Compared to UFH, the administration of its shortened derivatives, namely low-molecular-weight heparin (enoxaparin, nadroparin, dalteparin, tinzaparin and others), allows to reduce the risk of thrombocytopenia and hemorrhages [8,10]. Despite all the risks, the use of heparins is a standard therapeutic protocol. Therefore, in most preclinical and clinical trials, UFH is used as the comparator drug.

Nevertheless, it is the prevalence of heparin-induced thrombocytopenia among patients that has caused the demand for new effective anticoagulants. Hirudin-1 from the medicinal leech *Hirudo medicinalis* is the most studied among the direct thrombin inhibitors. A large number of different modified forms of this peptide have been developed and have been proven to have increased antithrombotic activity [11]. One of the recombinant analogues of hirudin-1, namely desirudin (known under the trade names Revasc, Canyon Pharmaceuticals, London, UK, or Iprivask, Bausch Health, Laval, QC, Canada), was once approved by the European Agency for the Evaluation of Medicinal Products (EMA) and the US Food and Drug Administration (FDA) for the treatment of heparin-induced thrombocytopenia and associated thrombotic disease but is currently withdrawn from circulation in the US [12,13]. Its recombinant derivative lepirudin (Refludan, Schering AG, Berlin-Wedding, Germany) with increased antithrombotic activity has also been completely withdrawn from circulation. Although lepirudin showed high efficacy and safety during three large clinical trials, called the Heparin-Associated-Thrombocytopenia (HAT) 1, 2, and 3, serious frequent hemorrhagic adverse events have been reported later, including death, limb amputation, bleeding from puncture sites and wounds, intracranial bleeding and other major bleeding [14,15].

Long-term studies of hirudin-1 and the mechanism of its inhibition of thrombin formed the basis for the creation of its shortened synthetic analogue, bivalirudin (Angiomax, Novartis AG, Basel, Switzerland) [16,17]. Bivalirudin has been certified for treatment of patients with, or at risk of, heparin-induced thrombocytopenia (HIT) or heparin-induced thrombocytopenia and thrombosis syndrome (HITTS) undergoing percutaneous coronary intervention and of patients with unstable angina pectoris after percutaneous transluminal coronary angioplasty [18,19]. Other areas of application are now being actively studied, although they have not received FDA approval. The studied application areas include the following: (1) as an addition to thrombolytic therapy of the acute myocardial infarction and for its prophylaxis after percutaneous coronary intervention; (2) for prophylaxis of the deep venous thrombosis; (3) administration during heart surgery against the background of heparin-induced thrombocytopenia; (4) for prophylaxis of the peripheral arterial bypass; (5) in therapy of the acute coronary syndrome patients undergoing percutaneous coronary intervention; (6) in therapy of the coronary percutaneous intervention (BRIGHT-4 trial); (7) for systemic anticoagulation during therapy of pediatric and adult patients requiring Extracorporeal Membrane Oxygenation [20,21,22,23,24,25]. Almost in all of these studies, bivalirudin shows very high efficacy and safety, which makes it a very promising anticoagulant.

The main disadvantages of drugs like bivalirudin are, firstly, the parenteral form of administration, which means they can only be used on an outpatient basis, and, secondly, the lack of antidotes to quickly eliminate the anticoagulant effect. With this research background taken into consideration, a significant amount of effort has been dedicated to the development of a whole group of highly specific direct anticoagulants with oral administration: dabigatran etexilate (Pradaxa, Boehringer Ingelheim Pharma, GmbH & Co. KG, Ingelheim, Germany), rivaroxaban (Xarelto, Bayer AG, Leverkusen, Germany), apixaban (Eliquis, Bristol Myers Squibb, Princeton, NJ, USA) and edoxaban (Lixiana, Daiichi Sankyo, Tokyo, Japan) [6]. Oral administration, combined with a long half-life in the bloodstream, greatly facilitates the long-term use of drugs for prophylactic purposes and eliminates the need for constant monitoring. A distinctive feature of dabigatran, or rather its prodrug dabigatran etexilate, is the existence of an antidote, idarucizumab (Praxbind, Boehringer Ingelheim Pharma, GmbH & Co. KG, Ingelheim, Germany), a fragment of a monoclonal antibody that allows you to quickly inactivate all traces of the drug circulating in the blood [26,27]. Dabigatran etexilate has received approval for use to reduce the risk of ischemic stroke and systemic embolism in nonvalvular atrial fibrillation, to treat deep venous thrombosis (DVT) and pulmonary embolism (PE), to prevent recurrent DVT and PE in adults (DVT/PE) and for primary prevention of venous thromboembolism in orthopedic surgery [28,29]. Moreover, after a clinical trial (RE-VERSE AD), idarucizumab was successfully approved to inactivate dabigatran etexilate [30]. In many studies, dabigatran etexilate is used as a comparator along with heparin.

Our research in recent years has been focused on the development of new direct anticoagulants from various blood-sucking organisms [31,32]. Since these drugs are experimental, their evaluation requires a comprehensive, reproducible and uniform approach that includes experiments in vitro and in vivo. Over the past decades of research, a large number of different animal models have been created to evaluate the antithrombotic potential and risks of pathological bleeding. We have selected the models that are easy to reproduce and adapt to existing laboratory conditions and that also allow us to obtain reliable results with little statistical scatter. We have chosen three anticoagulants as research subjects. Variegin from the tick *Amblyomma variegatum* was chosen as the experimental drug, while unfractionated heparin and dabigatran etexilate were chosen as comparator pharmaceuticals. Variegin is the smallest (32 aa) among the naturally occurring bivalent high-specificity thrombin inhibitors [33]. The main advantages of variegin are the high inhibition potency towards thrombin and the ability to retain activity after thrombin-associated degradation, resulting in more prolonged action. These facts, along with the high degree of homology between the C-terminal fragments of bivalirudin and variegin, allow us to hypothesize that the pharmacological and immunogenicity characteristics of these anticoagulants are similar. Therefore, variegin is a highly interesting and promising anticoagulant as the most similar counterpart to the highly effective pharmaceutical bivalirudin.

## 2. Results

### 2.1. Assessment of Pharmacodynamic Profile

**Figure 1:**
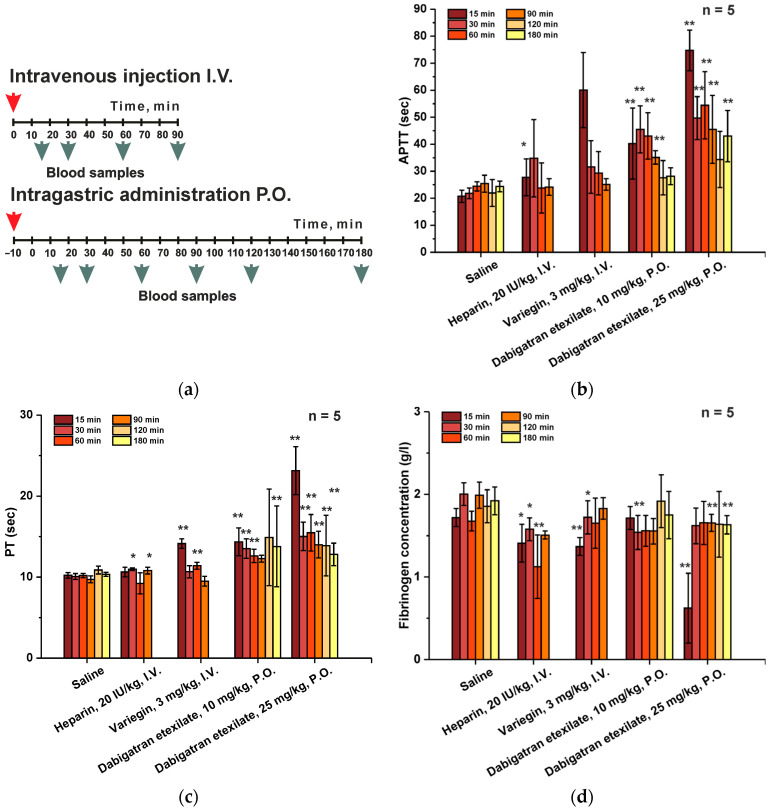
Assessment of pharmacodynamic profile. (**a**) Experiment design. Dynamics of APTT (**b**), PT (**c**), and fibrinogen concentration (**d**) in male ICR mice after administration of saline (5 mL/kg intravenously), heparin (10 IU/kg/5 mL intravenously), dabigatran etexilate (10 mg/kg/10 mL and 25 mg/kg/10 mL orally) and variegin (3 mg/kg/5 mL intravenously). Values are presented as mean ± SD (for each *n* = 5 point). * *p* ≤ 0.05, ** *p* ≤ 0.01 relative to saline group according to Mann–Whitney U-test.

The study of the pharmacodynamic profile is a primary task for the evaluation of any therapeutic drug. A classic method that reflects the effect of antithrombotic pharmaceuticals on blood coagulation processes is the measurement of hemostasis parameters (partial activated thromboplastin time (APTT), prothrombin time (PT) and fibrinogen concentration). The effect of drug administration on changes in these parameters was studied at different time points after administration (15, 30, 60, 90, 120 and 180 min, Figure 1a, Tables S1–S5, Supplementary Material). Since dabigatran etexilate was an oral anticoagulant, the experiment did not begin from the moment of its administration, but began only after 10 min, so that the drug could have sufficient time to be absorbed.

The APTT assessment is the standard practice in the evaluation of anticoagulants. Therefore, we have used this test for the investigation of the pharmacodynamics of pharmaceuticals (Figure 1b). In all groups, the introduction of drugs led to an increase in this parameter. In the group that received heparin, a very large variability in APTT values was observed (up to 42%), which does not allow us to correctly evaluate the results obtained from the sample of the five animals used. In general, with heparin at a dose of 20 IU/kg, APTT was increased by 1.3–1.5 times, but by 90 min after administration, the effect completely disappeared. The most significant time-dependent effect was observed in groups of animals that received variegin and dabigatran etexilate. The greatest effect (a 3-fold increase in APTT) was observed in the group that received variegin at 15 min after administration. However, by 30 min, it had decreased to a value exceeding control group only 1.5-fold, and continued to slowly decrease until there was no difference in comparison to the animals from the control group “Saline”. The effect of variegin completely disappeared after a time period close to 90 min. This fact indicates rapid elimination of the drug. The most significant effect was observed in the groups receiving dabigatran etexilate. At a dose of 10 mg/kg, dabigatran etexilate resulted in a 2-fold increase in APTT at 15 min (25 min after administration) and a 3.6-fold increase in APTT at a dose of 25 mg/kg. Subsequently, the effect slowly decreased. At the time the experiment was stopped (180 min), APTT exceeded the norm by 1.2 times at a dose of 10 mg/kg and 1.8 times at a dose of 25 mg/kg. It should be noted that there was significant variability in the results obtained in the groups that received variegin and dabigatran etexilate (from 10 to 35%), which indicates the need to increase the number of animals in these experiments.

PT measurement (Figure 1c) is not a test recommended for monitoring anticoagulants. There were no changes in this indicator in the group that received heparin. In the group that received variegin, there was a 1.4-fold increase in PT at 15 min after administration, after which it decreased to control values. In groups that received dabigatran etexilate at a dosage of 10 mg/kg, a similar increase in PT by 1.4 times was observed, but the effect persisted for 180 min until the end of the experiment. At a dosage of 25 mg/kg, dabigatran etexilate led to a 2-fold increase in PT at 15 min of the experiment, but by 30 min the effect decreased 1.4-fold relative to the norm and was maintained at this level until the end of the experiment. There was no significant effect of the drugs compared to saline on changes in fibrinogen concentrations (Figure 1d). Therefore, measurements of PTT and fibrinogen concentration cannot be used to assess the pharmacodynamics of study direct anticoagulants.

### 2.2. Rat Tail-Cut Bleeding Model

**Figure 2:**
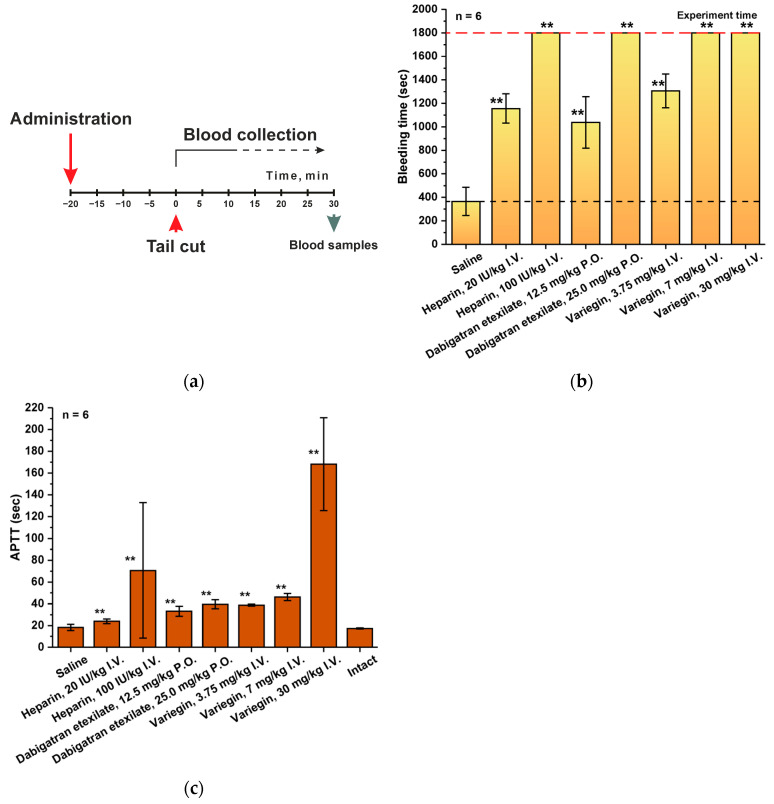
Rat Tail-Cut Bleeding Model. (**a**) Experiment design. (**b**) Tail-cut bleeding time in male SD rats after administration of saline (5 mL/kg intravenously (I.V.)), heparin (20 IU/kg/5 mL I.V. in group 2 and 100 IU/kg/5 mL I.V. in group 3), dabigatran etexilate (oral (P.O.), 12.5 mg/kg/10 mL in group 4 and 25 mg/kg/10 mL in group 5) and variegin (I.V., 3.75 mg/kg/5 mL in group 6, 7.5 mg/kg/5 mL in group 7 and 30 mg/kg/5 mL in group 8). ** *p* ≤ 0.01 compared to the saline group according to the Mann–Whitney test. The red dashed line indicates the total duration of the experiment (**c**) Dose-dependance dynamics of APTT at bleeding stop time. Values are presented as mean ± SD (for each group *n* = 6). ** *p* ≤ 0.01 compared to the saline group according to the Mann–Whitney test.

The tail bleeding model simulates the situation of uncontrolled bleeding or hemorrhage under the influence of an anticoagulant, since this is the main negative factor of any antithrombotic therapy. In the conditions of antithrombotic drug testing, the main criterion for the correctness of the experiment is the possibility of reliable control of the duration of bleeding, and, therefore, the selection of optimal and comparable working doses of the analyzed substances. In the initial experiments performed, the bleeding time in the groups of animals receiving heparin (a 100 IU/kg dose), dabigatran etexilate (a 25 mg/kg dose) and variegin (a 7 or 30 mg/kg dose) exceeded 30 min, which was the final goal of our study (Figure 2). Therefore, in further experiments, it was important not only to record the time of bleeding cessation, but also to select such concentrations of drugs at which the bleeding prolongation effect would be approximately the same. This is necessary for the correctness of drug effectiveness comparison. We have observed that the dosage of the experimental drug variegin of 3.75 mg/kg caused a statistically significant increase in the duration of bleeding by 3.6 times (1306 ± 143 s versus 365 ± 120 s for the group receiving saline), which is comparable to the effect of heparin at a dose of 20 IU/kg (1156 ± 125 s or 3.2-fold increase) and dabigatran etexilate at a dose of 12.5 mg/kg (1037 ± 220 s or 2.8-fold increase) (Table S6, Supplementary Material). It should be noted that the variability of results obtained using this model was in the range of 11–20%.

Control of bleeding time was also accompanied by estimation of coagulation times, and primarily APTT, as the main parameter for monitoring anticoagulants (Tables S7–S9, Supplementary Material). There were no significant differences in the measured values of APTT when the bleeding had stopped between the intact group and the saline group (Figure 2c). It is noteworthy that at the moment when the bleeding has ended, the APTT values for all groups that received anticoagulants was no more than 2 times higher than normal. The obtained APTT values correlate with the results obtained when assessing pharmacodynamics. It should be noted that a 2-fold increase in dosage for dabigatran etexilate and variegin had only a slight effect on the change in APTT, but at the same time led to prolonged bleeding.

A complete hematological analysis (Table S10, Supplementary Material) showed the absence of significant differences (variability does not exceed 10%) in all parameters at the time of bleeding control between the intact group, the group that was treated with saline, and the groups that were treated with anticoagulants.

### 2.3. Rat Inferior Vena Cava (IVC) Incomplete Stenosis Model of Venous Thrombosis Associated with Hypercoagulation

**Figure 3:**
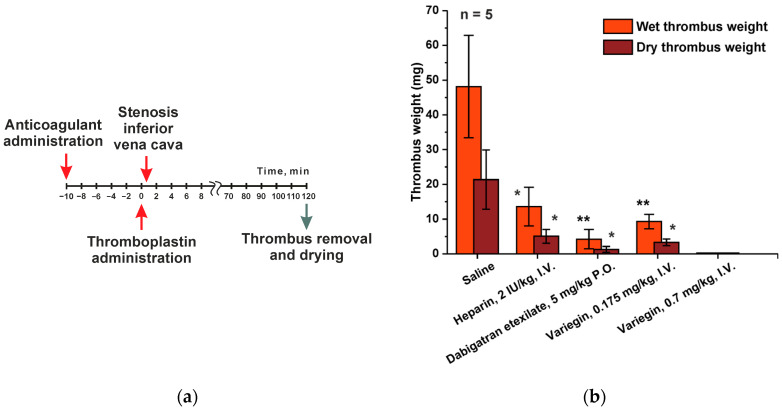
Inferior Vena Cava (IVC) Incomplete Stenosis Model of Venous Thrombosis. (**a**) Experiment design. (**b**) Wet and dried thrombus weights in male SD rats treated with saline 2 mL/kg (intravenously (I.V.)), heparin 2 IU/kg/2 mL (I.V.), dabigatran etexilate 5 mg/kg/2 mL (oral (P.O.)) and variegin 0.175 mg/kg/2 mL and 0.7 mg/kg/2 mL (I.V.). Values are presented as mean ± SEM (for each group *n* = 5). * *p* ≤ 0.05, ** *p* ≤ 0.01 compared to the saline group according to the Mann–Whitney test.

The main therapeutic purpose of anticoagulant drugs is to prevent the development of venous thrombosis. Therefore, the biological activity of these drugs is investigated on animal models where pathological thrombus formation is induced by thrombogenic compound administration, optionally in combination with physical surgery. From the wide variety of existing animal models for testing, we have selected the inferior vena cava (IVC) incomplete stenosis model of venous thrombosis associated with hypercoagulation. In this model, vein stenosis was caused by applying an occluder to narrow the vessel lumen and stimulate the development of thrombosis. While developing a method of induction of hypercoagulation pathological situation, we have chosen two approaches of extrinsic coagulation pathway activation. The first one is the injection of thromboplastin, which is one of the key thrombogenic stimuli. The second approach is the application of a piece of filter paper soaked in a solution of ferric chloride to the proximal portion of the vein, catalyzing of the lipid peroxidation. Since the model was intended for the study of anticoagulants, it was necessary to optimize several parameters of experimental protocol to guarantee the statistical reliability of the obtained results. The key evaluated criterion was the weight of the extracted thrombus (Figure 3).

We have optimized two parameters: the time from the moment of occlusion to the removal of the thrombus (from 30 min to 2 h), as well as the dosage of the thrombogenic agent, ranging from 0.0135 up to 0.054 ISI/kg for thromboplastin, or from 10× to 2.5× dilution of reagent Renamplastin (RENAM, Moscow, Russia). The “ISI/kg” unit is defined as the ratio of the International Sensitivity Index (which is calculated relative to the International WHO reference for thromboplastin and listed in the reagents specifications) to the mass of the experimental animal. The results of the experiments comparing the two methods discussed above suggest that the weight of the thrombus formed due to thromboplastin administration was two times larger compared to the one obtained using induction with ferric chloride. The results of the negative control (without administration of an anticoagulant) were the most stable when the experiment time exceeded 60 min, while the dose of the administered thromboplastin was 0.054 ISI/kg. Therefore, the anticoagulant effect of all studied drugs was tested under these conditions (Figure 3a).

As expected, there was a significant reduction in thrombus weight after administration of all anticoagulants (Figure 3b). We have decided to increase the time from the moment of occlusion to 120 min for simplification of blood clot removal. Investigation into the effect of anticoagulant administration on the “wet thrombus weight” indicator has revealed a 3.5-fold decrease for heparin (2 IU/kg dose), 11.5-fold for dabigatran etexilate (5 mg/kg dose) and 5.2-fold for variegin (0.175 μg/kg dose) (Table S11, Supplementary Material). As for the “dry thrombus weight” indicator (with the same anticoagulant doses), the observed decrease was 4.2-fold for heparin, 16.7-fold for dabigatran etexilate and 6.4-fold for variegin.

## 3. Discussion

Preclinical studies are an integral stage for the introduction of any new pharmaceutical compound. Regardless of its purpose, the validity of the interpretation of the obtained results is determined primarily by the correctness of the biological model choice and the reproducibility of the investigation approach. The development of a new effective antithrombotic agent involves finding a balance between high effectiveness in preventing blood clots and minimal risk of pathological bleeding. Only in this case can such an anticoagulant be successfully used in the preventive and therapeutic practice of thrombo-associated diseases. We have adapted various previously reported methods for the investigation of different anticoagulants. The comparative study benchmark that we have developed will allow us to perform the complete and comprehensive screening of anticoagulant activity for a set of thrombin inhibitors of our interest (haemadin from the leech *Haemadipsa sylvestris*, variegin from the tick *Amblyomma variegatum* and anophelin from *Anopheles albimanus*) in comparison with unfractionated heparin and dabigatran etexilate, which are already widely used in therapy [31,32].

During preclinical studies of any pharmaceutical drug, one of the most important factors is the assessment of the compounds pharmacokinetic and/or pharmacodynamic parameters. The obtained data makes it possible to investigate the drugs half-life, bioavailability and stability, and subsequently to use this information to build models for characterizing of the analyzed substances. The most important part of our investigation was the determinization of the duration of the physiological effect of the drugs on the animal’s body, which was performed by measuring of hemostasis parameters. These parameters include partial activated thromboplastin time (APTT), prothrombin time (PT), thrombin time (TT) and fibrinogen concentration at different time points after administration. Results of the PT and APTT investigation play a critical role, since studying the dynamics of these parameters allows to assess the blood coagulation disorders both along the general pathway and separately according to extrinsic coagulation tissue factor pathway (by PT) or by the intrinsic contact system pathway (according to APTT). The main parameter for direct thrombin inhibitors monitoring is the APTT measurement, while PT is more suitable for indirect anticoagulants (vitamin K antagonists) testing. Although we have carried out TT measurements, we did not include them in the reported results, since they were only qualitative in nature. In the study, the TT value was maintained within the reference range. The changes in fibrinogen concentration were monitored for the same purposes.

All of the investigated drugs have caused APTT prolongation. It was important for us to determine the drug dosages, within which APTT prolongation was proportional. As a result, it was found that in the heparin group at the study dosage of 20 IU/kg, an extremely high result variability was observed. Based on the observed effects, it is correct to compare variegin at a dosage of 3 mg/kg and dabigatran etexilate at a dosage of 25 mg/kg–APTT. In both cases, APTT was prolonged more than three times. Nevertheless, while the effect of variegin administration completely disappeared by the end of the experiment, the effect of dabigatran etexilate remained at a level exceeding the norm 2-fold. This is due to the different types of drug administration and mechanisms of compound elimination in body. In general, it was the APTT results that were used in further experiments to monitor the effects of the investigated drugs. Other parameters, including PPT, TT and fibrinogen concentration were only measured as a reference to exclude pathologically high-dosage administration.

For many pharmaceutical substances, effective dosages are selected to ensure a longer duration of the effect. In the case of anticoagulants, this approach is not appropriate since the excessive anticoagulant effect is accompanied by an extremely high risk of developing pathological bleeding. Investigation of this factor includes modeling a of vessel (vein or artery) damage situation with subsequent study of the drug administration effect on bleeding stop time. The tail-cut bleeding model in rats is the most common and easily reproducible modification of variation of this approach. Despite the simplicity of the experiment, the results obtained are significantly affected by parameters such as position of the tail (horizontal or vertical), the environment surrounding the wound (air or saline), temperature, anesthesia and the method of injury [34,35]. We have tried to take into account the results of various previous studies and unify the methodology as much as possible. The purpose of the experiment was to determine the working drug dosages that result in a comparable duration of bleeding upon administration. As a result, we have discovered the dosages that are optimal for this investigation of pharmacodynamics: 3-fold prolongation of bleeding time was observed in the groups of heparin at a dose of 20 IU/kg, variegin at a dose of 3.75 mg/kg and dabigatran etexilate at a dose of 12.5 mg/kg. Larger dosages (even 2-fold) have caused unacceptably prolonged bleeding, the stopping of which could not be observed within the experiments timeframe. Notably, increasing doses had minimal effect on APTT.

The key experiment in the study of anticoagulants activity is to evaluate their effectiveness against pathological thrombus formation. Since the generally accepted strategy for using anticoagulants is the prevention and treatment of venous thromboembolism, it is correct to use animal models of venous thrombosis for the initial assessment of the investigated compounds. The modern theory of thrombus formation is based on the so-called “Virchow triad”, which refers to the slowing of blood flow (stasis), hypercoagulation and damage to the vascular wall [36]. Thus, these are the three main factors that are responsible for thrombogenesis.

Experimental models use various methods to induce these states separately or in different combinations: stenosis, stasis, vessel wall injury (mechanical, electrical, chemical, photochemical, laser-light), insertion of a foreign surface or injection of a prothrombotic factor [37]. Two factors are crucial for the experiment: the choice of animal subject and the vessel in which thrombus formation is to be induced. Rodents are the most commonly used animals to model various types of pathologies. This is explained by the physiological similarity of the functioning of the circulatory system with the human one; they are convenient for various surgical procedures and are relatively economically feasible to maintain. In most rodent models, the inferior vena cava is selected, as it is the most easily available. However, while stimulating thrombus formation in this vessel, one should consider the variability of the side- and back-branch anatomy that can influence thrombotic outcomes. As an alternative, acute thrombosis studies can be performed on the jugular, femoral or saphenous veins.

Typically, venous thrombosis is modeled in the context of an induced a hypercoagulable state. This is achieved through administration of various thrombogenic factors such as human serum, thromboplastin, activated prothrombin complex, factor Xa or recombinant tissue factor. As an alternative stimulus, thrombus formation and platelet aggregation can be induced by the disruption of the endothelial surface of the vessel through chemical, temperature or electrolytic damage or catheter insertion. In order to test the investigated drugs, we have chosen the model of inferior vena cava partial stenosis with the hypercoagulability being caused either by the administration of thromboplastin or as a result of chemical damage to the vessel with a ferric chloride solution. The use of thromboplastin to induce hypercoagulation turned out to be a more convenient technique, since it was easier to manipulate, which allowed to obtain larger clots. This, in turn, simplified the final assessment and allowed us to obtain more stable results. All of the studied agents displayed high efficacy in this model. In the presence of tested drugs the size of the resulting thrombus was significantly reduced compared to the control group. Little statistical variability was shown across all groups. Thus, we have proven the possibility of using this model in our laboratory conditions for the testing of all potential antithrombotic agents.

## 4. Materials and Methods

### 4.1. Reagents

The model drugs heparin (high molecular weight heparin, 5000 IU/mL, Moscow Endocrine Plant, Moscow, Russia) and dabigatran etexilate (Pradaxa, 150 mg capsules, Boehringer Ingelheim Pharma, GmbH & Co. KG, Ingelheim, Germany) were purchased from their respective commercial suppliers. The experimental drug, namely recombinant variegin from the tick *Amblyomma variegatum* with a pharmaceutical quality, was produced by us according to the protocol published previously [31].

### 4.2. Animals

All of the laboratory animals used in this study were sexually mature young male rats and mice without deviations in health. Among these rodents were 79 male Wistar rats aged 6–8 weeks, weighing 280–340 g and 120 male ICR mice aged 8–12 weeks, weighing 23–30 g. The animals were obtained from the nursery of laboratory animals “Pushchino”, Pushchino, Russia. Animals were acclimatized for at least 7 days, and after a clinical examination, they were divided into groups so that the weight of the animals within the groups did not differ by more than 10%. Animals were assigned numbers, which were indicated by punctures in the auricles. Mice were housed in type 3 cages (length × width × height: 425 × 266 × 155 mm, floor area: 820 cm^2^), 5 individuals per cage. Rats were kept in type 4 cages (length × width × height: 595 × 380 × 200 mm, floor area: 1820 cm), 2 individuals per cage. During the study, the animals were kept under controlled environmental conditions in a barrier zone with a “clean” and “dirty” corridor system. The temperature was kept in the range of 20–24 °C, relative humidity was 30–55%. The 12 h light cycle included two phases: “day” 08:00–20:00 and “night” 20:00–08:00. The air volume in the room was changed 10-fold per hour. SNIFF RI/M-H V1534-30 (Ssniff Spezialdiaten GmbH, Soest, Germany) complete granular rodent food was autoclaved and fed ad libitum. As an enrichment of the habitat, the animals were housed in plastic cages from red plastic (Tecniplast Mouse House, Buguggiate, Italy).

### 4.3. Drugs Administration

The administration of saline solution, heparin and variegin was carried out intravenously into the lateral tail vein. The animal was fixed in a special container house, the tail was heated in warm water (temperature 35–37 °C), after which the drug was slowly injected (a volume of 5 mL/kg) into the lateral tail vein using a syringe with the G26 needle.

Dabigatran etexilate was administered orally by gavage. The animal was fixed in a position with hand restraints, and a metal gavage with a round, smooth tip with a diameter of 1.5 mm for mice and 2.5 mm for rats was inserted orally. A syringe with a suspension of dabigatran etexilate aqueous solution in a volume of 10 mL/kg was attached to the gavage.

### 4.4. Assessment of Pharmacodynamic Profile

To study the dynamics of hemostasis parameters, 120 male ICR mice were used. Animals were divided into 3 groups of 20 animals (groups 1, 2 and 3) and 2 groups of 30 animals (groups 4 and 5) according to the scheme in Table 1. In each group, the animals were divided into subgroups of 5 animals, from which blood was taken 15, 30, 60, 90 min after saline or tested anticoagulants administration. In groups 4 and 5, additional subgroups of animals were identified, from which blood was taken after 120 and 180 min after saline or tested anticoagulants administration.

Blood sampling was carried out terminally in animals anesthetized with a 1:3 mixture of Telazol (Zoetis Manufacturing & Research, Girona, Spain) and Xylazine (Nita-Farm, Moscow, Russia) 500 µL/kg, with a separate group of five animals used for each time point. To obtain plasma, blood was placed in test tubes with 1% sodium citrate, centrifuged at 4 °C and 3000 rpm for 15 min, then the resulting plasma was taken and immediately measured partial activated thromboplastin time (APTT), prothrombin time (PT) and fibrinogen concentration.

### 4.5. Tail-Cut Bleeding Model

A tail-cut bleeding model in male SD rats was used to study the clotting time in peripheral vascular injury. A total of 54 males were used, which were divided into 9 groups according to the scheme in Table 2.

**Figure 4:**
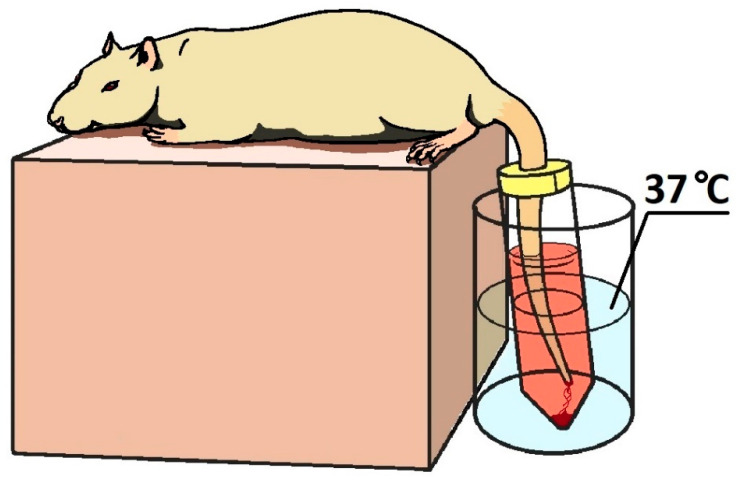
Illustration of the simulation of tail bleeding in rats. The anesthetized animal is placed on an elevated platform, the tail with the tip cut off is placed in a thermostated vessel with saline solution.

Tail-cut bleeding-model experiments were performed on anesthetized animals. Anesthesia was performed with Telazol:Xylazine: mixture 1:3. At 20 min before the start of the experiment (cutting off the tip of the tail), the animals were given drugs in accordance with the group affiliation (Table 2). In anesthetized animals, the tip of the tail was cut off with a blade at a distance of 3 mm from the end of the tail. The damaged tail was placed in a in a 50 mL transparent vessel with saline solution thermostated at 37 °C (Figure 4). The vessel was sealed with parafilm from above with a puncture for the rats tail. The tail was half-immersed in saline solution. The bleeding time was recorded for 30 min. All the described procedures were performed on groups of intact animals, with the exception of the administration of any drug and cutting off the tail. Blood samples were taken terminally to measure of partial activated thromboplastin time (APTT), prothrombin time (PT) and fibrinogen concentration and for hematological analysis.

### 4.6. Rat Inferior Vena Cava (IVC) Incomplete Stenosis Model of Venous Thrombosis

A model of incomplete inferior vena cava stenosis in male SD rats was used to assess the effect of the ability of drugs to reduce thrombus formation. A total of 25 males were used, which were divided into 5 groups according to the scheme in Table 3.

**Figure 5:**
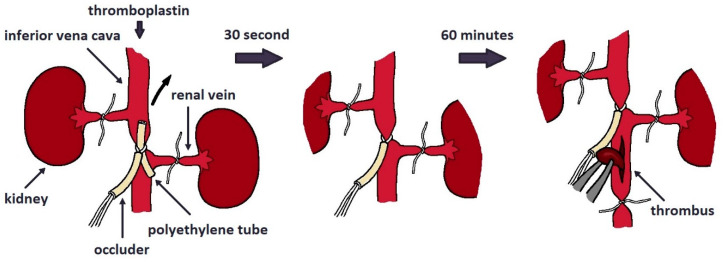
Schematic illustration of modeling venous thrombosis using the method of incomplete stenosis of the inferior vena cava.

The experiment was performed on animals anesthetized with the Telazol:Xylazine mixture 1:3. The animals received the saline or the tested anticoagulants before anesthesia. Anesthetized animals were placed on the operating table in the supine position, followed by an incision in the abdominal wall. Next, the intestines were moved back to provide free access to the vessels of the abdominal cavity. Both renal veins were ligated 10 min after the administration of the drug. Thromboplastin solution (Renamplastin, RENAM, Moscow, Russia, dissolved in 8 mL water and diluted 1:2.5 with saline) at a dose of 0.054 ISI/kg was injected intravenously in a volume of 1 mL 15 min after the administration of the saline or the tested anticoagulants. The occluder was tightened around the inferior vena cava in the area between the renal arteries 30 s after the injection of thromboplastin. The occluder was tightened around the vessel with a polyethylene catheter placed under it. After fixing the occluder loop around the vessel with the catheter placed under it, the catheter was removed from the loop, leaving a fixed lumen of the vessel, thus simulating incomplete stenosis of the inferior vena cava. After 120 min occlusion, the vessel was tied up 2 cm below the site of occlusion, the vessel wall was carefully opened with a longitudinal incision with ophthalmic scissors, and the thrombus was removed. The procedure is shown schematically in Figure 5. The thrombus was folded onto filter paper, blotted and placed in a pre-weighed Eppendorf tube. The Eppendorf tube with a thrombus was weighed, determining the mass of the wet thrombus. Then, the Eppendorf tube was opened and left at room temperature for 24 h, after which it was weighed again for determining the mass of the clot after drying.

### 4.7. Blood Sample Analysis

The parameters of hemostasis (APTT, PT and fibrinogen concentration) were determined using Renam reagents (Moscow, Russia).

Hematological analysis was performed on the day of blood sampling according to the parameters indicated in the Table 4 on a Mythic 18 Vet hematological analyzer (C2 DIAGNOSTICS S.A., Grabels, France).

### 4.8. Statistical Analyses

Statistica version 7.0 for Windows and GraphPad Prism version 8.0 software were used for statistical analysis. The results are expressed as mean values ± SD or mean values ± SEM. The number of mice and rats (N) used in the experiments are given in figure legends. The significance of differences in multiple comparisons was determined using one-way ANOVA and Mann–Whitney U-test. Significance level was determined at *p* ≤ 0.05.

## 5. Conclusions

In this research, we provide a comprehensive benchmark for the primary screening of new promising anticoagulants, which can be broadly applied regardless of their nature or mechanism of action. The animal models we have described allow to evaluate the pharmacodynamic characteristics of drugs and select working doses, which will be used to estimate their antithrombotic effect in comparison with standard drugs successfully used in therapeutic practice, such as unfractionated heparin and dabigatran etexilate. These approaches can be easily used to study drugs with different administration options and adapted to search for their specific antidotes.

We have tested our approach of comparative investigation of anticoagulant activity on two comparator drugs (heparin and dabigatran etexilate) and an experimental drug variegin from the tick *Amblyomma variegatum*. The experimental compound variegin is a direct anticoagulant and can be regarded as a natural analog of bivalirudin (Angiomax, Novartis AG, Basel, Switzerland). The study of variegin is very promising in light of the significant number of successfully completed and ongoing clinical studies of bivalirudin as an alternative to heparin in antithrombotic therapy of patients with heparin-induced thrombocytopenia.

## Figures and Tables

**Table 1:** Hemostasis model—groups and doses.

Group	Subgroup (for Each Subgroup *n* = 5)	Drug and Route of Administration	Dose and Volume of Administration	Time Point after Injection, min
1	1	Saline	5 mL/kg	15
	2			30
	3			60
	4			90
2	5	Heparin, intravenously	20 IU/kg, 5 mL/kg	15
	6			30
	7			60
	8			90
3	9	Variegin, intravenously	3 mg/kg, 5 mL/kg	15
	10			30
	11			60
	12			90
4	13	Dabigatran etexilate, peroral	10 mg/kg, 10 mL/kg	15
	14			30
	15			60
	16			90
	17			120
	18			180
5	19	Dabigatran etexilate, peroral	25 mg/kg, 10 mL/kg	15
	20			30
	21			60
	22			90
	23			120
	24			180

**Table 2:** Tail-cut bleeding model—groups and doses.

Group	Drug	Route of Administration	Dose and Volume of Administration
1 (*n* = 6)	Saline	Intravenously	5 mL/kg
2 (*n* = 6)	Heparin	Intravenously	100 IU/kg, 5 mL/kg
3 (*n* = 6)	Heparin	Intravenously	20 IU/kg, 5 mL/kg
4 (*n* = 6)	Dabigatran etexilate	Peroral	25 mg/kg, 10 mL/kg
5 (*n* = 6)	Dabigatran etexilate	Peroral	12.5 mg/kg, 10 mL/kg
6 (*n* = 6)	Variegin	Intravenously	30 mg/kg, 5 mL/kg
7 (*n* = 6)	Variegin	Intravenously	7.5 mg/kg, 5 mL/kg
8 (*n* = 6)	Variegin	Intravenously	3.75 mg/kg, 5 mL/kg
9 (*n* = 6)	Intact	-	-

**Table 3:** Venous thrombosis—groups and doses.

Group (for Each Group *n* = 5)	Drug	Route of Administration	Dose and Volume of Administration
1	Saline	Intravenously	2 mL/kg
2	Heparin	Intravenously	2 IU/kg, 2 mL/kg
3	Dabigatran etexilate	Peroral	5 mg/kg, 2 mL/kg
4	Variegin	Intravenously	0.7 mg/kg, 2 mL/kg
5	Variegin	Intravenously	0.175 mg/kg, 2 mL/kg

**Table 4:** Parameters of hematological analysis.

Parameters of Hematological Analysis	
Leucocyte, 10^9^/L	Hemoglobin, g/L
Lymphocyte, 10^9^/L	Hematocrit, l/L
Monocyte, 10^9^/L	Mean cell volume, fl
Granulocyte, 10^9^/L	Mean cell hemoglobin, pg
Lymphocyte, %	Mean corpuscular hemoglobin concentration, g/L
Monocyte, %	RBC distribution width, %
Granulocyte, %	RDW standard deviation, fl
Red blood cell, 10^12^/L	Platelet count, 10^9^/L

## Data Availability

All raw data are available upon request from the corresponding author.

## Supplementary Materials

The following supporting information can be downloaded at: https://www.mdpi.com/article/10.3390/ijms25073986/s1.
