# Supplementary material for: Unified Methodology for the Primary Preclinical In Vivo Screening of New Anticoagulant Pharmaceutical Agents from Hematophagous Organisms
Source: Int J Mol Sci. 2024 Apr 3;25(7):3986. doi: 10.3390/ijms25073986 (PMC11011928; doi:10.3390/ijms25073986)
Supplement: Supplementary file 1 [file ijms-25-03986-s001.zip › ijms-2877405-supplementary.pdf]

Supplementary Material

Unified Methodology for the Primary Preclinical In Vivo Screening of New Anticoagulant Pharmaceutical Agents from Hematophagous Organisms

Maria A. Kostromina [1], Elena A. Tukhovskaya [2,*], Elvira R. Shaykhutdinova [2], Yuliya A. Palikova [2], Viktor A. Palikov [2], Gulsara A. Slashcheva [2], Alina M. Ismailova [2], Irina N. Kravchenko [2], Igor A. Dyachenko [2], Evgeniy A. Zayats [1], Yuliya A. Abramchik [1], Arkady N. Murashev [2] and Roman S. Esipov [1]

[1] Laboratory of Biopharmaceutical Technologies, Shemyakin and Ovchinnikov Institute of Bioorganic Chemistry, Russian Academy of Sciences, Miklukho-Maklaya Street, 16/10, 117997 Moscow, Russia; kostromasha@gmail.com (M.A.K.); eaz96post@gmail.com (E.A.Z.); ugama@yandex.ru (Y.A.A.); esipov@ibch.ru (R.S.E.)

[2] Biological Testing Laboratory, Branch of Shemyakin and Ovchinnikov Institute of Bioorganic Chemistry, Russian Academy of Sciences, Pushchino, ProspektNauki, 6, 142290 Moscow, Russia; shaykhutdinova@bibch.ru (E.R.S.); yuliyapalikova@bibch.ru (Y.A.P.); vpalikov@bibch.ru (V.A.P.); slashcheva_ga@mail.ru (G.A.S.); ismailowa.a.m@yandex.ru (A.M.I.); ikravchenko@bibch.ru (I.N.K.); dyachenko@bibch.ru (I.A.D.); murashev@bibch.ru (A.N.M.)

* Correspondence: tukhovsksya@bibch.ru

Pharmacodynamic profile after substance administration. Time-dependance effect on clotting times and fibrinogen concentration

Table S1. Individual values of hemostasis parameters in male ICR mice, saline solution, 5 ml intravenously

**Parameter**	**Saline solution, 15 min**						
**№ mice**	**1**	**2**	**3**	**4**	**5**	**Mean**	**SD**
APTT, sec	17.2	21.9	19.7	22.7	22.1	**20.7**	**2.3**
PTT, sec	9.9	10	10	10.6	10.6	**10.2**	**0.3**
Fibrinogen concentration, g/l	1.779	1.696	1.544	1.736	1.831	**1.717**	**0.109**

**Parameter**	**Saline solution, 30 min**						
**№ mice**	**6&**	**7**	**8**	**9**	**10**	**Mean**	**SD**
APTT, sec	-	20.9	22.3	19.7	24.3	**21.8**	**2.0**
PTT, sec	-	9.6	10.3	10	10.4	**10.1**	**0.4**
Fibrinogen concentration, g/l	-	2.039	2.164	1.838	1.964	**2.001**	**0.137**

**Parameter**	**Saline solution, 60 min**						
**№ mice**	**11**	**12**	**13**	**14**	**15**	**Mean**	**SD**
APTT, sec	24.7	23.5	25.4	22	26.4	**24.4**	**1.7**
PTT, sec	10.1	10.1	10.3	9.9	10.6	**10.2**	**0.3**
Fibrinogen concentration, g/l	1.646	1.518	1.838	1.736	1.634	**1.674**	**0.120**

**Parameter**	**Saline solution, 90 min**						
**№ mice**	**16**	**17**	**18**	**19**	**20&**	**Mean**	**SD**
APTT, sec	24.8	29	26.3	21.5	-	**25.4**	**3.1**
PTT, sec	9.8	10	10	9.1	-	**9.7**	**0.4**
Fibrinogen concentration, g/l	1.904	1.933	1.893	2.226	-	**1.989**	**0.159**

**Parameter**	**Saline solution, 120 min**						
**№ mice**	**21**	**22**	**23**	**24**	**25**	**Mean**	**SD**
APTT, sec	13.4	24.0	22.4	26.0	24.0	**22.0**	**5.0**
PTT, sec	10.3	10.5	10.9	11.5	11.2	**10.9**	**0.5**
Fibrinogen concentration, g/l	1.964	1.973	1.973	1.508	1.854	**1.854**	**0.200**

**Parameter**	**Saline solution, 180 min**						
**№ mice**	**26**	**27**	**28**	**29**	**30**	**Mean**	**SD**
APTT, sec	24.9	23.1	25.5	21.6	26.7	**24.4**	**2.0**
PTT, sec	10.5	10	10.6	10.2	10.4	**10.3**	**0.2**
Fibrinogen concentration, g/l	1.833	1.904	1.793	2.211	1.862	**1.921**	**0.167**

& Data not received for technical reasons

* p≤0.05, ** p≤0.01 relative to the "saline" group according to the Mann-Whitney U-test

Table S2. Individual values of hemostasis parameters in male ICR mice, heparin, 20 IU/kg/5 ml intravenously

Parameter	Heparin, 20 IU/kg, 15 min						
**№ mice**	**31**	**32**	**33**	**34**	**35**	**Mean**	**SD**
APTT, sec	31.7	37.5	20.4	23.8	25.2	**27.7 ***	**6.8**
PTT, sec	11.1	11.3	10.5	10.5	9.8	**10.6**	**0.6**
Fibrinogen concentration, g/l	1.42	1.698	1.535	1.286	1.104	**1.409 ***	**0.228**

Parameter	Heparin, 20 IU/kg, 30 min						
**№ mice**	**36**	**37**	**38[&]**	**39**	**40**	**Mean**	**SD**
APTT, sec	21.8	32.3	-	29.9	55.2	**34.8 ***	**14.3**
PTT, sec	10.8	11.2	-	11	10.9	**11.0**	**0.2**
Fibrinogen concentration, g/l	1.49	1.433	-	1.67	1.717	**1.578 ***	**0.137**

Parameter	Heparin, 20 IU/kg, 60 min						
**№ mice**	**41**	**42[&]**	**43**	**44**	**45**	**Mean**	**SD**
APTT, sec	15.2	-	21	37	21.9	**23.8**	**9.3**
PTT, sec	7.4	-	9.8	9.3	10.4	**9.2**	**1.3**
Fibrinogen concentration, g/l	1.202	-	1.291	0.568	1.44	**1.125 ***	**0.384**

Parameter	Heparin, 20 IU/kg, 90 min						
**№ mice**	**46**	**47**	**48**	**49[&]**	**50**	**Mean**	**SD**
APTT, sec	23.6	27.8	20.3	-	24.9	**24.2 ***	**3.1**
PTT, sec	10.5	11.2	10.4	-	11.1	**10.8**	**0.4**
Fibrinogen concentration, g/l	1.52	1.567	1.461	-	1.482	**1.508 ***	**0.047**

& Data not received for technical reasons

* $p \leq 0.05$, relative to the “saline” group according to the Mann-Whitney U-test

Table S3. Individual values of hemostasis parameters in male ICR mice, variegin, 3 mg/kg/5 ml intravenously

Parameter	Variegin, 3 mg/kg, 15 min						
**№ mice**	**51**	**52**	**53**	**54**	**55**	**Mean**	**SD**
APTT, sec	84.4	56.6	49.8	52.9	56.6	**60.1 ****	**13.9**
PTT, sec	14.2	15.1	13.5	14.1	13.8	**14.1 ****	**0.6**
Fibrinogen concentration, g/l	1.292	1.268	1.544	1.381	1.360	**1.369 ****	**0.108**

Parameter	Variegin, 3 mg/kg, 30 min						
**№ mice**	**56**	**57**	**58**	**59**	**60**	**Mean**	**SD**
APTT, sec	44.1	36.8	18	28.5	30.6	**31.6**	**9.7**
PTT, sec	11.7	11.1	9.7	10.3	10.5	**10.7**	**0.8**
Fibrinogen concentration, g/l	1.878	1.389	1.75	1.878	1.709	**1.721 ***	**0.200**

Parameter	Variegin, 3 mg/kg, 60 min						
**№ mice**	**61**	**62**	**63**	**64**	**65**	**Mean**	**SD**
APTT, sec	23.4	38.8	18.9	31.8	33.6	**29.3**	**8.0**
PTT, sec	11	11.6	11.8	11.7	10.9	**11.4 ****	**0.4**
Fibrinogen concentration, g/l	1.588	1.45	2.175	1.437	1.599	**1.650**	**0.303**

Parameter	Variegin, 3 mg/kg, 90 min						
**№ mice**	**66**	**67[&]**	**68**	**69**	**70[&]**	**Mean**	**SD**
APTT, sec	22.8	-	25.4	27.1	-	**25.1**	**2.2**
PTT, sec	9.9	-	9.8	8.8	-	**9.5**	**0.6**
Fibrinogen concentration, g/l	1.684	-	1.862	1.937	-	**1.828**	**0.130**

* p≤0.05, ** p≤0.01 relative to the "saline" group according to the Mann-Whitney U-test

Table S4. Individual values of hemostasis parameters in male ICR mice, dabigatran etexilate (10 mg/kg/10ml oral)

Parameter	Dabigatran etexilate, 10 mg/kg, 15 min						
**№ mice**	**71**	**72**	**73**	**74**	**75**	**Mean**	**SD**
APTT, sec	31.5	23.9	56.4	39.9	49.5	**40.2 ****	**13.1**
PTT, sec	13.9	11.8	15	14.6	16.4	**14.3 ****	**1.7**
Fibrinogen concentration, g/l	1.767	1.841	1.698	1.777	1.482	**1.713**	**0.139**

Parameter	Dabigatran etexilate, 10 mg/kg, 30 min						
**№ mice**	**76**	**77**	**78**	**79**	**80**	**Mean**	**SD**
APTT, sec	49.6	36.5	39.9	43	58.5	**45.5 ****	**8.7**
PTT, sec	14.2	13.1	11.9	13.3	15.1	**13.5 ****	**1.2**
Fibrinogen concentration, g/l	1.652	1.248	1.777	1.583	1.433	**1.539 ****	**0.204**

Parameter	Dabigatran etexilate, 10 mg/kg, 60 min						
**№ mice**	**81**	**82**	**83**	**84**	**85**	**Mean**	**SD**
APTT, sec	41.3	31.9	42.3	43.8	55.9	**43.0 ****	**8.6**
PTT, sec	12	12.3	12.1	12.7	14	**12.6 ****	**0.8**
Fibrinogen concentration, g/l	1.505	1.374	1.863	1.583	1.468	**1.559**	**0.186**

Parameter	Dabigatran etexilate, 10 mg/kg, 90 min						
**№ mice**	**86**	**87**	**88**	**89[&]**	**90**	**Mean**	**SD**
APTT, sec	35.1	38	31.9	-	35.5	**35.1 ***	**2.5**
PTT, sec	12	12.9	12	-	12.2	**12.3 ***	**0.4**
Fibrinogen concentration, g/l	1.727	1.355	1.543	-	1.591	**1.554**	**0.154**

Parameter	Dabigatran etexilate, 10 mg/kg, 120 min						
**№ mice**	**91**	**92**	**93**	**94**	**95**	**Mean**	**SD**
APTT, sec	21.2	30.6	23	37	26.2	**27.6**	**6.4**
PTT, sec	20.1	10.7	10.3	10.8	22.6	**14.9**	**6.0**
Fibrinogen concentration, g/l	2.377	1.652	1.875	1.600	2.078	**1.916**	**0.320**

Parameter	Dabigatran etexilate, 10 mg/kg, 180 min						
**№ mice**	**96**	**97**	**98**	**99**	**100**	**Mean**	**SD**
APTT, sec	31.2	24.2	28	26.2	31.2	**28.2**	**3.1**
PTT, sec	11.9	10.9	11.1	22.6	12.4	**13.8**	**5.0**
Fibrinogen concentration, g/l	2.022	1.634	1.583	2.078	1.426	**1.749**	**0.286**

& Data not received for technical reasons

* p≤0.05, ** p≤0.01 relative to the “saline” group according to the Mann-Whitney U-test

Table S5. Individual values of hemostasis parameters in male ICR mice, dabigatran etexilate (25 mg/kg/10ml oral)

Parameter	Dabigatran etexilate, 25 mg/kg, 15 min						
**№ mice**	**101**	**102**	**103**	**104**	**105**	**Mean**	**SD**
APTT, sec	78.8	85.9	71.5	69.8	67.7	**74.7 ****	**7.5**
PTT, sec	24	27.2	24	20.8	19.7	**23.1 ****	**3.0**
Fibrinogen concentration, g/l	0.469	0.374	0.81	0.197	1.264	**0.623 ****	**0.422**

Parameter	Dabigatran etexilate, 25 mg/kg, 30 min						
**№ mice**	**106**	**107**	**108**	**109**	**110**	**Mean**	**SD**
APTT, sec	47.8	56.9	37.3	50	56.3	**49.7 ****	**8.0**
PTT, sec	14.5	17.6	13.6	13.5	15.9	**15.0 ****	**1.7**
Fibrinogen concentration, g/l	1.667	1.421	1.97	1.521	1.514	**1.619**	**0.215**

Parameter	Dabigatran etexilate, 25 mg/kg, 60 min						
**№ mice**	**111**	**112**	**113**	**114**	**115**	**Mean**	**SD**
APTT, sec	45.9	47.7	47.5	55.2	75.8	**54.4 ****	**12.5**
PTT, sec	14.5	14.1	14.1	15.2	19.4	**15.5 ****	**2.2**
Fibrinogen concentration, g/l	1.842	1.915	1.684	1.579	1.254	**1.655**	**0.260**

Parameter	Dabigatran etexilate, 25 mg/kg, 90 min						
**№ mice**	**116**	**117**	**118**	**119**	**120**	**Mean**	**SD**
APTT, sec	30.9	46.1	41.1	43.8	65.4	**45.5 ****	**12.6**
PTT, sec	13.7	13.1	13.1	13.2	16.9	**14.0 ****	**1.6**
Fibrinogen concentration, g/l	1.659	1.684	1.793	1.618	1.507	**1.652**	**0.104**

Parameter	Dabigatran etexilate, 25 mg/kg, 120 min						
**№ mice**	**121**	**122**	**123**	**124**	**125**	**Mean**	**SD**
APTT, sec	38.1	37.5	35.2	44.2	16.7	**34.3**	**10.4**
PTT, sec	11.9	11.9	12.1	13	20.5	**13.9 ****	**3.7**
Fibrinogen concentration, g/l	1.243	1.505	1.626	1.512	2.299	**1.637**	**0.396**

Parameter	Dabigatran etexilate, 25 mg/kg, 180 min						
**№ mice**	**126**	**127**	**128**	**129**	**130**	**Mean**	**SD**
APTT, sec	35.7	33.3	41.4	56.7	47.8	**43.0 ****	**9.5**
PTT, sec	11.7	11.2	12.8	14.5	14	**12.8 ****	**1.4**
Fibrinogen concentration, g/l	1.757	1.679	1.661	1.461	1.591	**1.630 ****	**0.111**

** p≤0.01 relative to the “saline” group according to the Mann-Whitney U-test

Table S6. Tail-cut bleeding time after substance administration in male SD rats (individual values)

Number of animals in the group	Saline	Heparin, 20 IU/kg, I.V.	Heparin, 100 IU/kg, I.V.	Dabigatran etexilate, 12.5 mg/kg P.O.	Dabigatran etexilate, 25 mg/kg P.O.	Variegin, 3.75 mg/kg, I.V.	Variegin, 7.5 mg/kg, I.V.	Variegin, 30 mg/kg, I.V.
1	252	1101	1800	1095	1800	1511	1800	1800
2	586	953	1800	959	1800	1338	1800	1800
3	376	1164	1800	691	1800	1217	1800	1800
4	343	1185	1800	950	1800	1113	1800	1800
5	268	1331	1800	1299	1800	1247	1800	1800
6	366	1203	1800	1230	1800	1409	1800	1800
**Mean**	**365**	**1156 ****	**1800 ****	**1037 ****	**1800 ****	**1306 ****	**1800 ****	**1800 ****
**SD**	**120**	**125**	**0**	**220**	**0**	**143**	**0**	**0**

** p≤0.01 relative to the "saline" group according to the Mann-Whitney U-test

Table S7. Individual values of APTT after substance administration in male SD rats in tail-cut bleeding time (individual values)

Number of animals in the group	Saline	Heparin, 20 IU/kg, I.V.	Heparin, 100 IU/kg, I.V.	Dabigatran etexilate, 12.5 mg/kg P.O.	Dabigatran etexilate, 25 mg/kg P.O.	Variegin, 3.75 mg/kg, I.V.	Variegin, 7.5 mg/kg, I.V.	Variegin, 30 mg/kg, I.V.	Intact animal
1	15.1	22.3	14.6	37.3	43.7	39	49	145.4	17.8
2	17	25.3	16	27.2	40.4	39.9	47.2	184.8	17.1
3	20.3	22.3	111.7	27.6	37.3	38.5	42.6	106.5	17.1
4	20.8	27.5	100.4	36.4	44.9	37.1	ND	146	ND
5	15.3	22.5	20.2	35.6	34.2	39	ND	214.9	ND
6	21.2	23.6	160.5	34.8	36.7	38.7	ND	211.4	ND
**Mean**	**18.3**	**23.9 ****	**70.6**	**33.2 ****	**39.5 ****	**38.7 ****	**46.3 ***	**168.2 ****	**17.3**
**SD**	**2.8**	**2.1**	**62.2**	**4.5**	**4.2**	**0.9**	**3.3**	**42.8**	**0.4**

* p≤0.05, ** p≤0.01 relative to the "saline" group according to the Mann-Whitney U-test

Table S8. Individual values of PTT after substance administration in male SD rats in tail-cut bleeding time (individual values)

Number of animals in the group	Saline	Heparin, 20 IU/kg, I.V.	Heparin, 100 IU/kg, I.V.	Dabigatran etexilate, 12.5 mg/kg P.O.	Dabigatran etexilate, 25 mg/kg P.O.	Variegin, 3.75 mg/kg, I.V.	Variegin, 7.5 mg/kg, I.V.	Variegin, 30 mg/kg, I.V.	Intact animal
1	19.5	21.3	18.9	27.2	38.7	29.4	31.5	101.2	22.4
2	20.2	21.4	19.9	25.0	34.5	31.0	35.7	161.9	23.3
3	19.9	20.5	24.1	24.8	30.4	30.9	30.3	79.1	21.0
4	20.4	23.4	24.3	28.0	34.7	29.2	ND	100.9	ND
5	19.3	22.0	20.0	28.3	27.1	28.8	ND	212.4	ND
6	20.1	23.3	29.6	28.8	34.8	29.4	ND	208.8	ND
**Mean**	**19.9**	**22.0 ****	**22.8**	**27.0 ****	**33.4 ****	**29.8 ****	**32.5 ***	**144.1 ****	**22.2 ***
**SD**	**0.4**	**1.2**	**4.0**	**1.7**	**4.0**	**0.9**	**2.8**	**58.5**	**1.2**

* $p \leq 0.05$, ** $p \leq 0.01$ relative to the "saline" group according to the Mann-Whitney U-test

Table S9. Individual values of fibrinogen concentration after substance administration in male SD rats in tail-cut bleeding time (individual values)

Number of animals in the group	Saline	Heparin, 20 IU/kg, I.V.	Heparin, 100 IU/kg, I.V.	Dabigatran etexilate, 12.5 mg/kg P.O.	Dabigatran etexilate, 25 mg/kg P.O.	Variegin, 3.75 mg/kg, I.V.	Variegin, 7.5 mg/kg, I.V.	Variegin, 30 mg/kg, I.V.	Intact animal
1	2.377	1.933	1.646	1.97	1.626	1.868	1.619	0.967	1.922
2	2.197	1.9	1.683	2.091	2.078	1.777	1.740	0.739	1.994
3	2.053	1.991	1.816	2.299	2.254	3.001	1.603	1.053	2.003
4	2.299	1.596	1.665	2.078	1.701	1.956	ND	0.912	ND
5	2.531	1.956	1.665	2.041	2.029	1.900	ND	0.486	ND
6	2.377	2.003	1.588	2.053	1.701	1.816	ND	0.5	ND
**Mean**	**2.306**	**1.897 ****	**1.677 ****	**2.089 ***	**1.898 ***	**2.053 ***	**1.654 ***	**0.776 ****	**1.973 ***
**SD**	**0.165**	**0.152**	**0.076**	**0.111**	**0.256**	**0.469**	**0.075**	**0.242**	**0.044**

* $p \leq 0.05$, ** $p \leq 0.01$ relative to the "saline" group according to the Mann-Whitney U-test

Table S10. Results of hematological analysis in male SD rats in tail-cut bleeding time

Parameters	Saline	Heparin, 20 IU/kg, I.V.	Dabigatran etexilate, 12.5 mg/kg P.O.	Dabigatran etexilate, 25 mg/kg P.O.	Variegin, 3.75 mg/kg, I.V.	Variegin, 7.5 mg/kg, I.V.	Intact animal
	n=6	n=6	n=6	n=6	n=3	n=3	n=3
Leucocyte, 10^9/l	10.6 ± 1.6	13.4 ± 3.2	9.4 ± 0.6	9.8 ± 1.8	9.7 ± 1.8	8.5 ± 1.3	10.8 ± 1.9
Lymphocyte, 10^9/l	9.2 ± 1.5	12.1 ± 2.7	8.3 ± 0.6	8.6 ± 1.6	8.3 ± 1.7	7.5 ± 1.1	9.7 ± 1.6
Monocyte, 10^9/l	0.5 ± 0.1	0.4 ± 0.2	0.3 ± 0.1	0.3 ± 0.1	0.4 ± 0.1	0.3 ± 0.1 *	0.4 ± 0.1
Granulocyte, 10^9/l	0.9 ± 0.1	0.9 ± 0.5	0.8 ± 0.1	0.9 ± 0.2	1,0 ± 0.2	0.7 ± 0.1	0.8 ± 0.2
Lymphocyte, %	87,0 ± 1.5	90.4 ± 2,0	88.5 ± 0.4	87.5 ± 1.4	85.4 ± 2.9	88.3 ± 0.8	89.2 ± 0.7
Monocyte, %	4.5 ± 0.3	3.2 ± 0.5	3.4 ± 0.4	3.1 ± 0.4	4,0 ± 0.8	4,0 ± 0.4	3.3 ± 0.4
Granulocyte, %	8.5 ± 1.3	6.4 ± 1.5	8.1 ± 0.6	9.4 ± 1.6	10.6 ± 2.6	7.7 ± 0.5	7.5 ± 0.3
Red blood cell, 10^12/l	8.69 ± 0.32	8.90 ± 0.26	9.18 ± 0.12	8.83 ± 0.19	8.65 ± 0.36	8.63 ± 0.66	9.21 ± 0.33
Hemoglobin, g/l	165 ± 4	159 ± 7	161 ± 5 *	159 ± 2 *	159 ± 9	157 ± 13 *	166 ± 6
Hematocrit, l/l	0.472 ± 0.017	0.483 ± 0.023 **	0.494 ± 0.016	0.479 ± 0.004	0.472 ± 0.027	0.463 ± 0.041 **	0.504 ± 0.016
Mean cell volume, fl	54.3 ± 0.5	54.2 ± 1.2 **	53.8 ± 1.8 *	54.3 ± 1,0 *	54.6 ± 1.1	53.6 ± 1.3 *	54.7 ± 0.4 *
Mean cell hemoglobin, pg	19,0 ± 0.4	17.8 ± 0.3 *	17.6 ± 0.4	18,0 ± 0.3	18.3 ± 0.4	18.2 ± 0.5	18,0 ± 0.3
Mean corpuscular hemoglobin concentration, g/l	350 ± 6	329 ± 6	327 ± 5 *	331 ± 2	336 ± 3	340 ± 6	329 ± 3
RBC distribution width, %	11.3 ± 0.2	12.9 ± 0.5	12.8 ± 0.5	12.5 ± 0,0	11.9 ± 0.3	11.8 ± 0.4	12.6 ± 0.5
RDW standart deviation, fl	28,0 ± 1,0	31.6 ± 2.1	32.3 ± 0,0	30.8 ± 1.4	29.6 ± 1,0	29.2 ± 1.3	31.6 ± 1.4 *
Platelets, 10^9/l	668 ± 25	736 ± 72	703 ± 42	735 ± 33	706 ± 29	671 ± 31	667 ± 20
Mean platelet volume, fl	5.3 ± 0.1	5.5 ± 0.2 **	5.5 ± 0.1 *	5.4 ± 0.1 *	5.4 ± 0.2 *	5.5 ± 0.2 *	5.5 ± 0.1 *
Plateletcrit, cl/l	0.354 ± 0.016	0.406 ± 0.041 **	0.389 ± 0.027 *	0.399 ± 0.016 *	0.379 ± 0.022 **	0.368 ± 0.028 *	0.369 ± 0.013 *
Platelet distribution width, %	14.7 ± 1.2	15.3 ± 1.1 **	15.9 ± 1.3 *	15.2 ± 0.6 *	14.1 ± 0.7 **	15.4 ± 0.9	16.0 ± 0.3 *

Data are presented as MEAN ± SD, * $p \leq 0.05$, ** $p \leq 0.01$ relative to saline group in accordance with Mann-Whitney U-test

Table S11. Inferior vena cava (IVC) stenosis model of venous thrombosis associated with hypercoagulation. Thrombus weights after substance administration in male SD rats (individual values)

**Number of animals in the group**	**Saline**		**Heparin, 2 IU/kg, I.V.**		**Dabigatran etexilate, 5 mg/kg P.O.**		**Variegin, 0.175 mg/kg, I.V.**	
**Thrombus**	**Wet**	**Dry**	**Wet**	**Dry**	**Wet**	**Dry**	**Wet**	**Dry**
1	84.4	43.2	1.0	0.0	15.0	4.5	6.2	1.7
2	35.5	12.5	6.5	2.6	0.0	0.0	5.0	1.9
3	57.0	0.0	7.8	3.7	3.0	0.5	14.7	6.1
4	15.7	25.6	21.3	7.9	0.0	0.0	6.5	1.9
5	0.0	4.1	31.4	11.1	3.0	1.4	14.0	5.0
**Mean**	**48.2**	**21.4**	**13.6 ***	**5.1 ***	**4.2 ****	**1.3 ***	**9.3 ****	**3.3 ***
SD	29.5	17.0	12.4	4.4	6.2	1.9	4.7	2.1
**SEM**	**14.7**	**8.5**	**5.6**	**2.0**	**2.8**	**0.8**	**2.1**	**0.9**

* $p \leq 0.05$, ** $p \leq 0.01$ relative to the “saline” group according to the Mann-Whitney U-test
